# Elucidating Best Geospatial Estimation Method Applied to Environmental Sciences

**DOI:** 10.1007/s00128-023-03835-0

**Published:** 2023-12-08

**Authors:** María de Lourdes Berrios Cintrón, Parya Broomandi, Jafet Cárdenas-Escudero, Jorge O. Cáceres, David Galán-Madruga

**Affiliations:** 1 Department of Health Sciences, Inter American University of Puerto Rico, Barranquitas Campus, Bo. Helechal Street 156, Barranquitas, Puerto Rico; 2 Department of Civil and Environmental Engineering, School of Engineering and Digital Sciences, Nazarbayev University, Kabanbay Batyr Ave. 53, Astana, 010000 Kazakhstan; 3 Analytical Chemistry Department, FCNET, University of Panama, University City, University Mail, Panama City, 3366 Panama; 4 Laser Chemistry Research Group, Department of Analytical Chemistry, Faculty of Chemistry, Complutense University of Madrid, Plaza de Ciencias 1, Madrid, 28040 Spain; 5 National Reference Laboratory of Air Quality, National Centre for Environmental Health (CNSA), Carlos III Health Institute (ISCIII), Ctra. Majadahonda a Pozuelo, Madrid, 28222 Spain

**Keywords:** Air Quality, PM_10_ Particles, Geostatistical Estimation, Interpolation Algorithms and Environmental Sciences

## Abstract

The aim of this study is to assess and identify the most suitable geospatial interpolation algorithm for environmental sciences. The research focuses on evaluating six different interpolation methods using annual average PM_10_ concentrations as a reference dataset. The dataset includes measurements obtained from a target air quality network (scenery 1) and a sub-dataset derived from a partitive clustering technique (scenery 2). By comparing the performance of each interpolation algorithm using various indicators, the study aims to determine the most reliable method. The findings reveal that the kriging method demonstrates the highest performance within environmental sciences, with a spatial similarity of approximately 70% between the two scenery datasets. The performance indicators for the kriging method, including RMSE (root mean square error), MAE (mean absolute error), and MAPE (mean absolute percentage error), are measured at 3.2 µg/m^3^, 10.2 µg/m^3^, and 7.3%, respectively.

This study addresses the existing gap in scientific knowledge regarding the comparison of geospatial interpolation techniques. The findings provide valuable insights for environmental managers and decision-makers, enabling them to implement effective control and mitigation strategies based on reliable geospatial information and data. In summary, this research evaluates and identifies the most suitable geospatial interpolation algorithm for environmental sciences, with the kriging method emerging as the most reliable option. The study’s findings contribute to the advancement of knowledge in the field and offer practical implications for environmental management and planning.

Environmental sciences encompass a broad range of disciplines that investigate the interrelationship between humans and the environment. These disciplines include ecology (Zhao 2023), climatology (Tu’uholoaki et al. 2022), environmental geochemistry (Deng et al. 2022), geology (Tadesse et al. 2023), and others, addressing various topics such as climate change (Deivanayagam et al. 2023), green building (Zhao et al. 2022), groundwater remediation (Beker et al. 2022), and air quality (Galán-Madruga 2021). Of particular significance within the field of environmental sciences is air quality, as atmospheric pollution poses a significant global environmental risk to human health (Madruga et al. 2019). Assessing human exposure levels to air pollutants is crucial in the context of public health. European legislation, such as Directive 2008/50/EC, emphasizes the importance of monitoring and controlling air quality through air quality networks comprising fixed monitoring stations to safeguard human health.

In this context, interpolation methods are widely employed to estimate human exposure levels in areas that have not been previously assessed. These methods facilitate spatial estimation and analysis, which play a crucial role in decision-making processes aimed at mitigating poor air quality conditions. Several research groups have utilized geospatial analysis to assess air quality in specific regions. For instance, Cardito et al. (2023) employed the inverse distance weighting tool to analyze the spatial distribution of six air pollutants and evaluate the impact of COVID-19 lockdown regulations on air pollution in Campania, Italy. Similarly, Broomandi et al. (2023) utilized the same interpolation algorithm to assess the health risks associated with metal-containing particulate matter in 158 European cities between 2013 and 2019, mapping the spatial correlation between potentially toxic elements and time. In another study, Kumar et al. (2020) investigated the influence of traffic-related air pollutants and associated risks along major transport corridors in Delhi. They utilized the kriging interpolation method to analyze air pollution levels and their spatial patterns. Galán-Madruga and García-Cambero (2022) focused on modeling benzene levels in an air quality network by considering other air pollutants and meteorological variables as predictor inputs. They applied the kriging interpolation technique to identify representative fixed stations within the target network.

While the previously mentioned research studies contribute valuable insights to scientific progress, they primarily focused on specific interpolation algorithms without evaluating a comprehensive range of methods for spatial interpolation. To address this gap, the current study aims to assess multiple conventional interpolation techniques commonly employed in geospatial estimation within environmental sciences. The goal is to provide robust evidence that identifies the most suitable interpolation method to be utilized in this field. By conducting a thorough analysis of various algorithms, this study aims to contribute to the advancement of geospatial estimation practices in environmental sciences.

## Materials and Methods

### Study Area and Reference Pollution Dataset

To achieve the proposed objective, this study was conducted in the Community of Madrid, located in the central region of the Iberian Peninsula. The Community of Madrid is home to an estimated population of over 6.7 million people and encompasses a land area of approximately 8,000 km^2^. It is comprised of 179 municipalities (INE 2022), making it a suitable area for investigation.

**Fig. 1:**
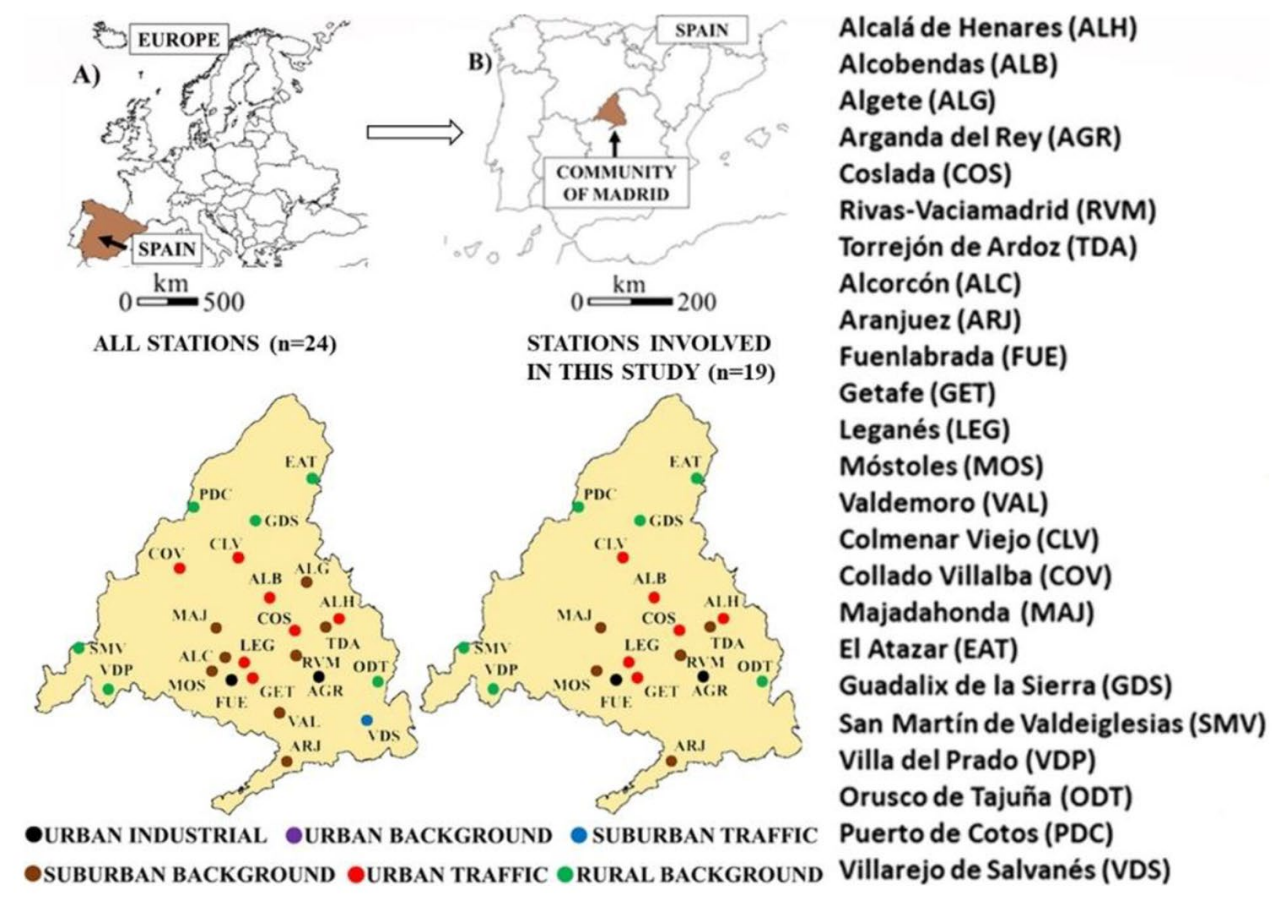
Location and type of fixed measurement stations belonging to the Community of Madrid’s AQMN

For this specific case study, the annual average PM_10_ concentrations in 2022 were examined rather than PM_2.5_ particles. Despite both atmospheric pollutants being included in the current European legislation, the limit values set for PM_10_ are stricter than for PM_2.5_ in terms of temporal scale. The legislation establishes a daily and annual average limit value for PM_10_ and only annual for PM_2.5_ (Directive 2008/50/EC). For this reason, PM_10_ particles were regarded for developing the present study. The PM_10_ concentrations were obtained from the fixed measurement stations included in the target air quality measurement network (AQMN) of the Community of Madrid. The measurement method for monitoring PM_10_ was beta absorption, and the equipment was an automatic analyzer. PM_10_ particles were chosen as they are known to be harmful to health and are subject to mandatory control by the European Union. During the investigation period, the AQMN in the Community of Madrid consisted of 24 fixed monitoring stations, with 79% of these stations measuring the target pollutant (as shown in Fig. 1). This constituted the reference dataset for the study, with a total of 19 stations included. The regional government was responsible for managing and ensuring the validity of the data obtained from the AQMN. In this regard, Directive 2008/50/EC (Annex I) sets data quality objectives for ambient air quality assessment to guarantee the validity of monitored data. For particulate matter, criteria such as 25% uncertainty and 90% minimum data capture should be complied.

### Proposed Approach: Background

In geospatial estimation methods, interpolation algorithms play a crucial role in providing unbiased information about values at non-sampled sites (Baume et al. 2011). To determine the most suitable method for environmental sciences, specifically in the context of air quality, the following sequence of steps was undertaken: (1) a sub-dataset was derived from a reference dataset by selecting fixed stations as sampling sites, (2) different interpolation algorithms were applied to the data from both the reference dataset (scenery 1) and the sub-dataset (scenery 2), and (3) the outcomes obtained from both scenery 1 and scenery 2 were evaluated to identify the best geospatial estimation method.

### Laying Down a Working Sub-Dataset

The initial step towards achieving the proposed objective involves creating a sub-dataset from the reference dataset using a scientifically established approach. In this study, a partitive clustering technique known as k-means clustering with a maximum of 10 iterations was utilized. This technique is commonly employed in data mining and aims to partition a set of n-observations (in this case, annual average PM_10_ levels) into k clusters, with each observation assigned to the group whose average value is most similar to it (Galán Madruga et al. 2018). The average value of each cluster is calculated by considering all observations within that cluster (Galán-Madruga et al. 2023).

Consistent with previous studies (Galán-Madruga et al. 2022), the Euclidean distance was used as an objective criterion to generate the clusters, serving as a spatial indicator. The study conducted nine clusters, varying the value of k from 2 to 10. The selection of the appropriate cluster required a coefficient of determination higher than 0.99 between the current annual average PM_10_ levels obtained from the reference dataset and those estimated by the clustering technique. The fixed stations within the selected cluster with less favorable Euclidean distance values were excluded, resulting in the remaining stations forming the working sub-dataset.

### Applying Various Interpolation Algorithms

This study evaluated six different interpolation algorithms, namely Inverse distance to the power, kriging, minimum curvature, nearest neighbor, radial basic function, and Shepard’s method. Each algorithm operates based on distinct principles to estimate values for non-measured data points. In the Inverse distance to the power method, the influence of one point relative to another decreases as the distance between them increases (Yang et al. 2023). Kriging calculates weighted averages of neighboring data points to determine non-measured values (Wang et al. 2023). The minimum curvature method assigns weights iteratively until changes in values are below a specified threshold (Ford and Moghrabi 1996). Nearest neighbor assigns the value of the nearest point to non-monitored data (Zaidi 2021). Radial basic function employs a weighted sum of radial basis functions to estimate non-measured values, encompassing various data interpolation techniques (Liu and Zhao 2022). Shepard’s method, on the other hand, represents the simplest form of inverse distance weighted interpolation (Dell’Accio et al. 2023).

To develop PM_10_ particle iso-concentration maps, Surfer for Windows (Win32) was utilized as a geographical information system (Surface Mapping System, v.6.04, Golden Software, Inc., Golden, CO, USA). Statistical analysis of the data was performed using IBM SPSS Statistics v29 (IBM Corp., Armonk, NY, USA).

### Appointing the Best Geospatial Estimation Method for Environmental Sciences

The selected interpolation algorithms were utilized to estimate PM_10_ concentrations in both scenery 1 (it corresponds to the original PM_10_ dataset) and scenery 2 (it corresponds to sub-dataset derived from original PM_10_ dataset). The comparison between the actual annual average PM_10_ concentrations of the removed stations and the estimated concentrations using the interpolation algorithms was conducted through simple linear regression analysis. Furthermore, the performance of the interpolation algorithms was assessed using indicators commonly employed in atmospheric sciences (Karunasingha 2022). These indicators include root mean square error (RMSE), mean prediction error (MPE), and mean absolute percentage errors (MAPE), which are calculated according to the equations provided by Dai et al. (2022).

## Results and Discussion

### Laying Down a Working Sub-Dataset

**Fig. 2:**
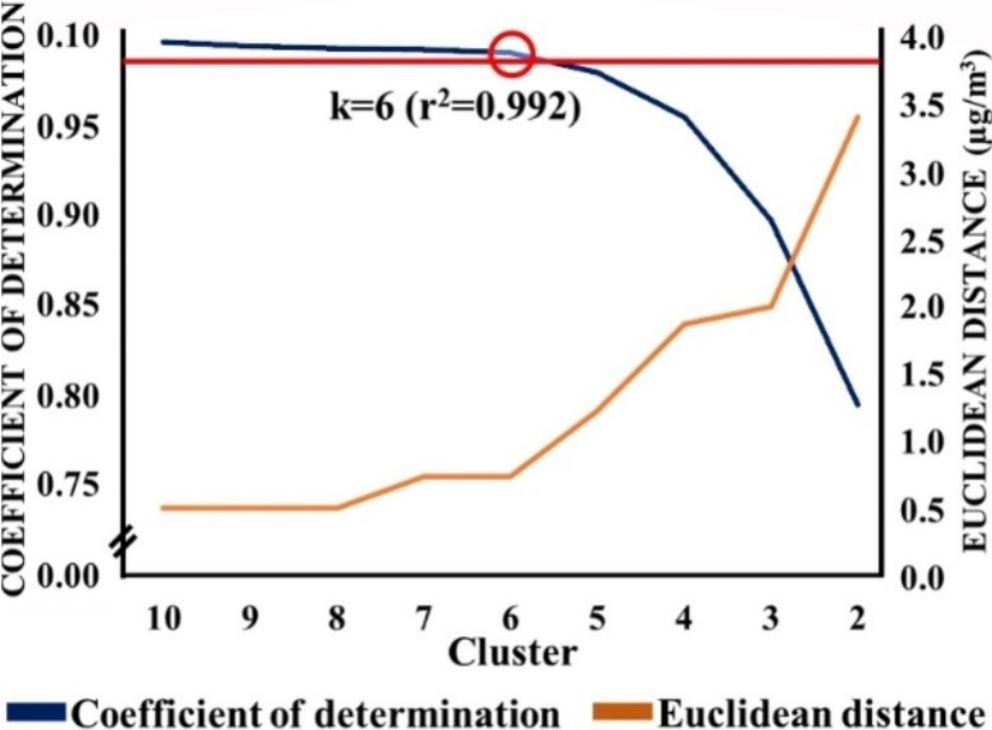
Outcomes resulting from k-means clustering analysis

Figure 2 illustrates the results obtained from the application of k-means clustering analysis to the reference dataset. The coefficient of determination, determined through a simple linear regression analysis between the current PM_10_ concentrations and those estimated by the clustering technique, ranged from 0.795 (CI: 0.737–0.958) to 0.998 (CI: 0.997-1.000) for clusters 2 and 10, respectively. It is important to note that as the number of clusters decreases, the coefficient of determination also diminishes. A coefficient of determination higher than 0.99 was considered as the selection criterion to identify the working cluster for further evaluation of the interpolation algorithms. Cluster 6 emerged as the first group with a coefficient of determination exceeding the established cutoff value (r^2^ = 0.992). Cluster 6 encompassed almost the entire information from the reference dataset, exhibiting a high level of similarity (> 99%). However, the Euclidean distance increased as the number of clusters decreased, resulting in a value of 0.733 µg PM_10_/m^3^ for cluster 6, equivalent to 6.75% expressed as relative data.

Once the working cluster (cluster 6) was determined, the subsequent step involved identifying the fixed stations that formed the working sub-dataset. To achieve this, the fixed stations within cluster 6 with the highest Euclidean distance for each sub-cluster (ranging from 1 to 6) were excluded. The remaining stations were selected to form the sub-working dataset, with the following stations being removed: FUE, COS, PDC, MAJ, SMV, and MOS, and the following stations being selected: ALH, CLV, ARJ, LEG, RVM, AGR, EAT, GDS, ODT, ALB, VDP, GET, and TDA (refer to Table 1 for details).

**Table 1:** Results obtained by running the k = 6 clustering analysis on reference dataset

			Annual average PM_10_ level	
Location	Group within cluster 6	Euclidean distance (µg/m^3^)	Current ^a^ (µg/m^3^)	Estimated ^b^ (µg/m^3^)
ALH	6	0.033	19.9	19.9
CLV	6	0.033	19.9	19.9
**FUE**	**6**	**0.067**	**19.8**	**19.9**
ARJ	5	0.325	22.9	23.2
**COS**	**5**	**0.475**	**23.7**	**23.2**
LEG	5	0.275	23.5	23.2
RVM	5	0.425	22.8	23.2
AGR	4	0.367	10.5	10.9
EAT	4	0.367	10.5	10.9
**PDC**	**4**	**0.733**	**11.6**	**10.9**
GDS	3	0.400	13.8	14.2
**MAJ**	**3**	**0.500**	**14.7**	**14.2**
ODT	3	0.100	14.1	14.2
ALB	2	0.067	16.6	16.7
**SMV**	**2**	**0.633**	**17.3**	**16.7**
VDP	2	0.567	16.1	16.7
GET	1	0.100	21.6	21.5
**MOS**	**1**	**0.500**	**21.0**	**21.5**
TDA	1	0.400	21.9	21.5

Key: ^a^ Concentration measured by the fixed PM_10_ stations ^b^ Concentration estimated by applying the k = 6 clustering analysis. Note: Fixed stations with the highest Euclidean distance per group are marked in bold. These are not included in the working sub-dataset, comprised by the stations non-marked

### Applying Interpolation Algorithms and Appointing the Most Suitable One

**Fig. 3:**
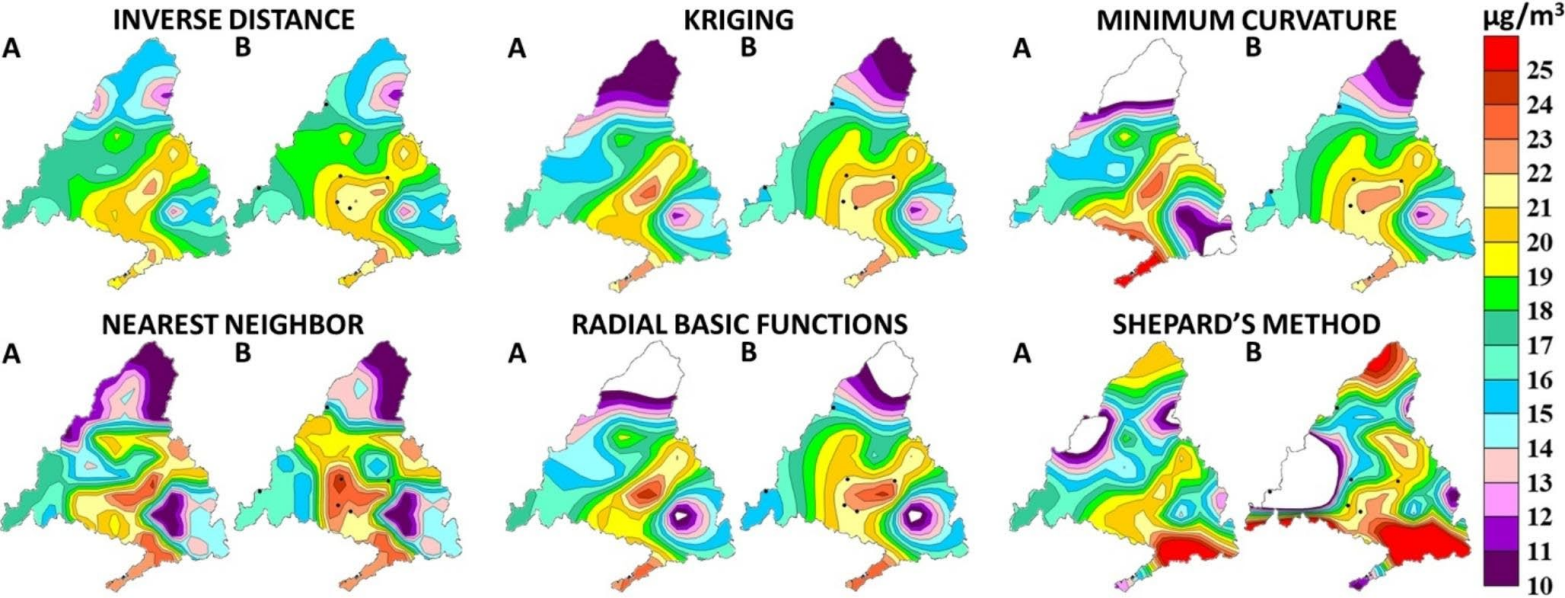
Annual average PM_10_ particles iso-concentration maps in 2022. (A) Map represented with the reference dataset (scenery 1, n = 19), and (B) Map represented with the working sub-dataset (scenery 2, n = 12)

Various interpolation algorithms were evaluated for geospatial estimation. Figure 3 illustrates the spatial distribution of PM_10_ gradients based on annual average concentrations from the reference dataset and the working sub-dataset, using each applied interpolation algorithm. Generally, there is a noticeable similarity in spatial representation between scenery 1 and 2 for most interpolation techniques. However, the minimum curvature algorithm stands out as it exhibits significantly different gradients, making it unsuitable for further evaluation within the scope of the study. Similarly, Shepard’s method is excluded from the assessment because it is unable to interpolate concentration levels for the six previously removed fixed stations, specifically SMV.

To determine the most suitable geospatial estimation technique in the field of environmental sciences, the Pearson’s coefficient of correlation was calculated between the current annual average PM_10_ levels at the removed fixed stations and the estimated values obtained from different interpolation algorithms. The calculated correlation coefficients were as follows (in ascending order): 0.204 for the nearest neighbor method, 0.602 for inverse distance to the power, 0.624 for radial basis function, and 0.697 for kriging. To interpret these results, the categorization proposed by Dancey and Reidy (2007) was applied, which classifies the degree of association between variables into five categories based on the correlation coefficient value: zero (0), weak (± 0.1–0.3), moderate (± 0.4–0.6), strong (± 0.7–0.9), and perfect (± 1).

Among the evaluated interpolation algorithms, inverse distance to the power and radial basis function demonstrates a moderate level of association, while kriging exhibits a strong level of association. On the other hand, the nearest neighbor technique shows a weak connection and is not suitable for the proposed objective of this study. To further confirm this finding, the bias between the current and estimated annual average PM_10_ levels at the removed stations was calculated. The average bias values were 15.3%, 14.9%, and 14.3% for inverse distance, radial basis function, and kriging, respectively. Performance indicators such as root mean square error (RMSE), mean absolute error (MAE), and mean absolute percentage error (MAPE) were also evaluated. The outcomes for inverse distance were 3.6 µg/m^3^, 13.3 µg/m^3^, and 8.2% for RMSE, MAE, and MAPE, respectively. For radial basis function, the values were 3.5 µg/m^3^, 12.3 µg/m^3^, and 8.1%. Lastly, kriging resulted in 3.2 µg/m^3^, 10.2 µg/m^3^, and 7.3% for RMSE, MAE, and MAPE, respectively. Based on the evidence gathered, the kriging method is considered the most suitable geospatial estimation technique for applications in environmental sciences.

Shukla et al. (2020) utilized kriging and inverse distance weighting as interpolation methods to generate particulate matter distribution maps in the megacity of Delhi. They reported an average error of 22% for kriging and 24% for inverse distance weighting. While the performance of these algorithms was slightly lower compared to the findings of this study, qualitatively, they also identified kriging as the superior spatial interpolation algorithm.

The relevance of geospatial analysis is sustained in providing (i) solutions to complex issues (Ahasan et al. 2022; Tadese et al. 2022), knowledge of scientific information in terms of geographics (Saldias et al. 2022), studying patterns (Kang et al. 2021), and conducting trend analysis and predictions (Liu et al. 2020). Given its wide application in environmental research, it is crucial to determine the most suitable interpolation method that can generate reliable outcomes for specific geospatial estimation processes. The procedure developed in this study fills the gap in scientific knowledge by comparing different geospatial interpolation techniques used in various environmental sciences, thus providing a robust body of evidence to identify the best interpolation method.

In conclusion, the findings presented in this study have important implications for environmental management, as geospatial information serves as a fundamental basis for decision-making (Hoang Tu et al. 2023). The results of this work can benefit research groups worldwide that require the application of spatial interpolation algorithms in their studies, facilitating the development of control plans, implementation of mitigation strategies, and informed decision-making by environmental managers.
